# X-Ray Phase-Contrast Tomography of Renal Ischemia-Reperfusion Damage

**DOI:** 10.1371/journal.pone.0109562

**Published:** 2014-10-09

**Authors:** Astrid Velroyen, Martin Bech, Irene Zanette, Jolanda Schwarz, Alexander Rack, Christiane Tympner, Tanja Herrler, Claudia Staab-Weijnitz, Margarita Braunagel, Maximilian Reiser, Fabian Bamberg, Franz Pfeiffer, Mike Notohamiprodjo

**Affiliations:** 1 Chair of Biomedical Physics, Department of Physics (E17), Munich, Bavaria, Germany; 2 Medical Radiation Physics, Lund University, Lund, Sweden; 3 European Synchrotron Radiation Facility, Grenoble, France; 4 Institute of Pathology, Ludwig-Maximilians-University Munich, Munich, Germany; 5 Department of General, Trauma, Hand, and Plastic Surgery, Ludwig-Maximilians-University Hospital Munich, Munich, Germany; 6 Institute for Clinical Radiology, University Hospitals Munich, Munich, Germany; 7 Comprehensive Pneumology Center, University Hospital, Ludwig-Maximilians-University and Helmholtz Zentrum Munich, Munich, Germany; 8 Department of Radiology, University Hospital Tuebingen, Tuebingen, Germany; Charité Universitätsmedizin Berlin, Germany

## Abstract

**Purpose:**

The aim of the study was to investigate microstructural changes occurring in unilateral renal ischemia-reperfusion injury in a murine animal model using synchrotron radiation.

**Material and Methods:**

The effects of renal ischemia-reperfusion were investigated in a murine animal model of unilateral ischemia. Kidney samples were harvested on day 18. Grating-Based Phase-Contrast Imaging (GB-PCI) of the paraffin-embedded kidney samples was performed at a Synchrotron Radiation Facility (beam energy of 19 keV). To obtain phase information, a two-grating Talbot interferometer was used applying the phase stepping technique. The imaging system provided an effective pixel size of 7.5 µm. The resulting attenuation and differential phase projections were tomographically reconstructed using filtered back-projection. Semi-automated segmentation and volumetry and correlation to histopathology were performed.

**Results:**

GB-PCI provided good discrimination of the cortex, outer and inner medulla in non-ischemic control kidneys. Post-ischemic kidneys showed a reduced compartmental differentiation, particularly of the outer stripe of the outer medulla, which could not be differentiated from the inner stripe. Compared to the contralateral kidney, after ischemia a volume loss was detected, while the inner medulla mainly retained its volume (ratio 0.94). Post-ischemic kidneys exhibited severe tissue damage as evidenced by tubular atrophy and dilatation, moderate inflammatory infiltration, loss of brush borders and tubular protein cylinders.

**Conclusion:**

In conclusion GB-PCI with synchrotron radiation allows for non-destructive microstructural assessment of parenchymal kidney disease and vessel architecture. If translation to lab-based approaches generates sufficient density resolution, and with a time-optimized image analysis protocol, GB-PCI may ultimately serve as a non-invasive, non-enhanced alternative for imaging of pathological changes of the kidney.

## Introduction

Renal ischemia-reperfusion (I/R) accounts for the majority of acute kidney injury cases [1], which often progress to chronic kidney disease [2]. Its consequences such as cortical and tubular necrosis are commonly encountered after renal transplantation [3] and form a potential basis for development of renal fibrosis, which is the final path of most renal diseases [4].

Clinically ill patients present with a decrease of renal function, i.e. glomerular filtration, however, there are no established laboratory tests for a reliable diagnosis of ischemia-reperfusion damage, so that renal biopsy remains the diagnostic standard of reference with all its risks and potential complications [5].

Morphological imaging modalities such as ultrasonography, computed tomography and magnetic resonance imaging are not ideally suited for detection of renal parenchymal disease, as early changes rather affect the ultrastructure and function than gross morphology [6]. Functional techniques such as perfusion and diffusion weighted MR imaging may reveal microstructural and functional changes [7]–[9], but spatial resolution is low [10], acquisition is not standardized and analysis is heavily influenced by the post-processing method [11].

X-ray phase-contrast imaging (PCI) and dark-field imaging (DFI) techniques, which exploit the refraction and small-angle scattering of x-rays in the investigated tissue in addition to their absorption, provide a novel contrast and complementary information [12]–[14] and thus may form a potential modality for non-enhanced, non-invasive kidney imaging [15]. The majority of PCI-studies on biological tissue have been performed at synchrotron radiation sources [16] which provide monochromatic x-rays with high flux and are used in combination with high-resolution imaging systems. PCI of the kidney without the use of a contrast agent has so far only been performed using crystal-based phase imaging techniques [15], [17]–[19]. These studies already demonstrate the innovative potential of this technique to depict the smallest anatomical structures and detect parenchymal renal disease such as glomerulosclerosis in hamster kidneys [15]. However, both the applied crystal-interferometer technique [17] as well as the analyzer-crystal based diffraction enhanced imaging (DEI) require a monochromatic, highly collimated x-ray beam. Consequently, their transfer from highly brilliant synchrotron sources to conventional x-ray sources suffers from long exposure times [20] and does not allow for the clinically widely used cone-beam geometry, thus rendering a transfer to a clinical setting difficult. Grating-based (GB) Talbot interferometry [21], [22] was successfully implemented in laboratory-based settings using conventional polychromatic x-ray sources by introduction of a third grating [13]. In synchrotron studies, it has proven excellent soft-tissue contrast and high-resolution volumetric data of various biomedical samples [23], [24]. Several studies on biomedical samples conducted at polychromatic x-ray sources have shown improved imaging of parenchymal lung disease [25], breast lesions [26], [27], cartilage [28], and atherosclerotic plaques [29] at good density resolution and reasonable spatial resolution.

The aim of this study was to investigate microstructural changes occurring in parenchymal renal disease, e.g. acute unilateral ischemic kidney injury in a murine animal model, using GB-PCI at a synchrotron radiation source. We hypothesize that GB-PCI allows depiction of volumetric and microstructural changes as well as vessel imaging without the use of contrast media.

## Materials and Methods

### Animals

Two male Balb/C nude (nu/nu) mice, aged 10–12 weeks and weighing 21–24 g, purchased from Charles River Laboratories (Sulzfeld, Germany) were included in this study. Animals were fed a standard diet and allowed free access to water. All animal experiments were conducted in accordance with institutional guidelines and were approved by the Administrative Panel on Laboratory Animal Care (AZ 55.2-1-54-2532-19-11, Government of Upper Bavaria, Germany).

### Renal Ischemia-Reperfusion Injury Model

Mice were anesthetized by intraperitoneal injection of a combination of 0.05 mg/kg fentanyl, 0.5 mg/kg medetomidine (Pfizer, Berlin, Germany), and 5 mg/kg midazolam (Ratiopharm, Ulm, Germany) and placed on a heated surgical pad to keep the body temperature constant. The right kidney was exposed through median abdominal incision, and mice were subjected to ischemia by clamping the renal pedicle with a nontraumatic microaneurysm clamp (Braun, Melsungen, Germany), which was removed after 45 min. The incision was closed with a 5–0 suture (Ethicon, Livingston, Scotland, UK) and surgical staples (Hugo Sachs GmbH, March, Germany). Postoperatively, anesthesia was antagonized by subcutaneous injection of a combination of 2.5 mg/kg atipamezole (Pfizer), 0.5 mg/kg flumazenil (Delta Select, Pfullingen, Germany), and 1.2 mg/kg naloxone (Inresa, Bartenheim, France). Mice were sacrificed by combined cervical dislocation and exsanguination. Tissue specimens of the ischemic right and the contralateral nonischemic kidney were collected on day 18 for histological analysis and embedded in paraffin according to standard protocols.

### GB-PCI Setup and Image Acquisition

**Figure 1:**
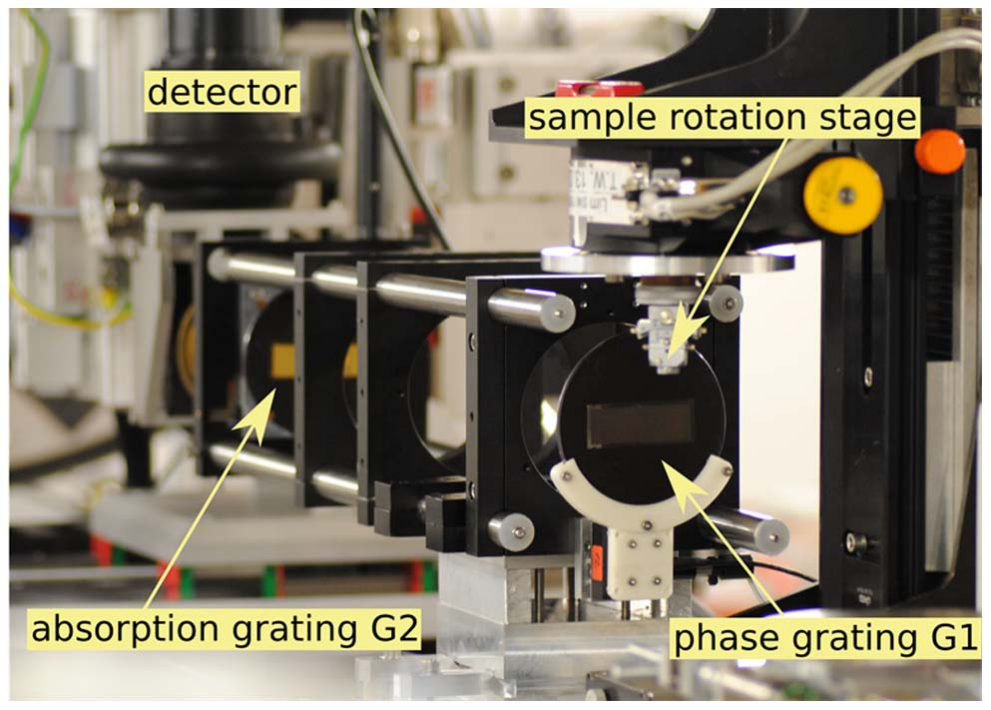
Photograph of the Interferometer Setup at Beamline ID19, ESRF, Grenoble, France. Monochromatic, coherent X-rays travel through the sample and introduce distortions in the interference pattern created by the phase grating. Those are evaluated by a lateral movement of the phase grating located behind the sample. Photograph adapted from I. Zanette, *Interférometrie X à réseaux pour l'imagerie et l'analyse de front d'ondes au synchrotron*, PhD thesis, University of Grenoble, 2011.

GB-PCI of the paraffin-embedded kidney samples was performed at beam line ID19 of the European Synchrotron Radiation Facility (ESRF), Grenoble, France. To extract the phase information of the x-ray beam passing through the sample, a two-grating Talbot interferometer was used [30], which was operated at a photon energy of 19 keV with an inter-grating distance corresponding to the 11th fractional Talbot order to assure high sensitivity. The periods and heights of the π-shifting silicon phase grating G1 and the gold analyzer grating G2 were p_1_ = 4.78 µm, h_1_ = 21 µm, and p_2_ = 2.4 µm, h_2_ = 100 µm, respectively. For x-ray detection the standard system at ID19, consisting of a Gadox scintillator coupled via lenses to a charge-coupled device camera, was used, providing an effective pixel size of 7.5 µm. A photograph of the setup is shown in **Figure 1**.

For image acquisition, the samples, embedded in paraffin blocks, were placed in an Eppendorf vial (Sigma Aldrich, St. Louis, USA). In order to ensure complete wetting of the paraffin surface and avoid trapping of air bubbles that cause image artifacts, a non-polar fluid, i.e. oil, was used to fill up the vial. To avoid phase-wrapping artifacts, the sample container was additionally placed into a box-shaped water basin. Tomographies of all four samples were acquired using the phase stepping procedure (as described earlier [21]) with 4 phase steps per projection and an exposure time of 5 seconds per step. Accounting for their difference in size, 701 projections were taken of the control kidneys, whereas only 501 projections of the clamped kidneys were acquired, each over 360 degrees.

### Post-Processing

The raw projection data was processed according to the standard phase stepping Fourier processing [12], [21]. The resulting attenuation, differential phase and dark-field projections were tomographically reconstructed using standard filtered back-projection, with a Ram-Lak (for attenuation and dark field) and a Hilbert filter (for phase data). To obtain quantitative volumetric data, the three-dimensional tomograms were segmented using the commercial software VGStudio MAX 2.0 (Volume Graphics, Heidelberg, Germany). Because the expenditure of time for this procedure is about 40 to 50 hours per dataset, the segmentation was only performed exemplarily on the dataset of one contralateral control and one I/R kidney. On the I/R dataset rough regions of interest were defined manually beforehand, in which the more precise segmentation was then performed by a combination of region growing, thresholding and manual segmentation. In the control kidney dataset the borders between the compartments appeared clearly defined, so that segmentation was mainly performed using thresholding, region-growing algorithms and occasional manual segmentation. The juxtacortical outer stripe of the outer medulla could not be distinguished from the cortex in the control kidney and not from the inner stripe in the I/R dataset.

### Histology

The specimens were stained with hematoxylin-eosin stain, iron stain, and Periodic acid-Schiff reaction for protein for conventional histopathologic evaluation. The slides were examined by an experienced uropathologist (C.T., with 7 years pathology experience). Histology was evaluated with respect to increased cellularity, disturbance of cellular polarity, failure of differentiation from the base to the surface, irregularity in the size of cells, variations of shape, protein cylinders and loss of brush borders and infiltration of leukocytes. A radiologist (M.N., with 7 years urogenital radiology experience), a physicist (A.V.), and the above-mentioned pathologist assessed the correlation between the PCI and histopathology findings in consensus.

### Statistical Analysis

Statistical analysis was performed with IBM SPSS Software 20 (IBM, Armonk, New York, USA). The gray values were extracted from the respective three-dimensional segmentations of the phase-contrast volumes of each dataset. A low quantity represents low electron density and vice versa. Due to the enormous data size (>100 million data points), statistical analysis was performed using the “random sampling” function, randomly selecting 10 000 datapoints from each kidney compartment. Significant differences between the gray values of the compartments were assessed with paired t-tests. Significance was assumed at *p*<0.05.

## Results

### Kidney Morphology

**Figure 2:**
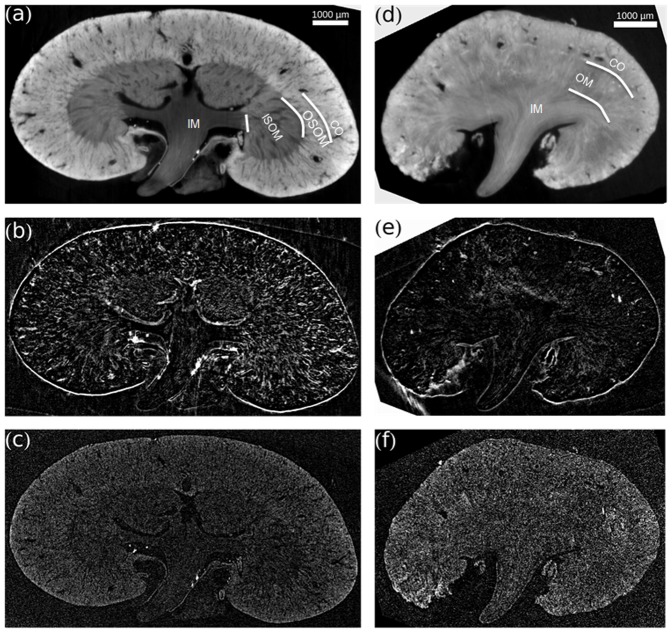
Contralateral Control Kidney and Ischemic Kidney in three Different Contrast Modalities. (a), (d) Phase contrast. (b), (e) Dark-field contrast. (c), (f) Attenuation contrast. All images are scaled individually for best visual appearance. (a) Phase-contrast images of the contralateral kidney allow to differentiate the functional compartments of the kidney. (d) Phase-contrast images of I/R kidneys show considerably less variation in gray value between the different compartments. Stripes of the outer medulla cannot be distinguished. The number of visible tubular structure or voids is highly reduced. In dark-field contrast, (b) control kidneys show scattering at interfaces, which is reduced in (e) I/R kidneys. Conventional absorption-based images show considerably lower contrast and relatively higher noise in both (c) control and (f) ischemic kidney. CO – cortex; OM – outer medulla; OSOM – outer stripe outer medulla; ISOM – inner stripe outer medulla; IM – inner medulla.

A qualitative overview of exemplary slices of the contralateral non-ischemic control kidney and the I/R kidney on day 18 in all three different contrast modalities (phase contrast, dark-field, attenuation) is provided in **Figure 2**. Phase-contrast images of the contralateral kidney (**Figure 2a**) allowed to differentiate the functional compartments of the kidney. Inner medulla, inner stripe of the outer medulla and cortex were clearly distinguishable by decreasing gray values. The outer stripe of the medulla showed gray values similar to the cortex. Also, an abundance of tubular structures and blood vessels was visible. In dark-field contrast images of control kidneys (**Figure 2b**) scattering at tubule-to-tissue interfaces was detected. The conventional absorption-based images (**Figure 2c**) exhibited a considerably lower contrast and relatively higher noise than the phase-contrast images. Phase-contrast images of I/R kidneys (**Figure 2d**) showed considerably less variation in gray values between the different compartments. Also, the tissue appeared hypotrophic and the number of visible tubular structures was markedly reduced. The outer stripe of the outer medulla showed a dramatic change in gray values after ischemia-reperfusion injury and could not be distinguished from the inner stripe. Correspondingly, in dark-field images the number of scattering structures was reduced in injured kidneys (**Figure 2e**). In the absorption-based images no additional information could be obtained (**Figure 2f**).

### Quantitative Analysis

**Figure 3:**
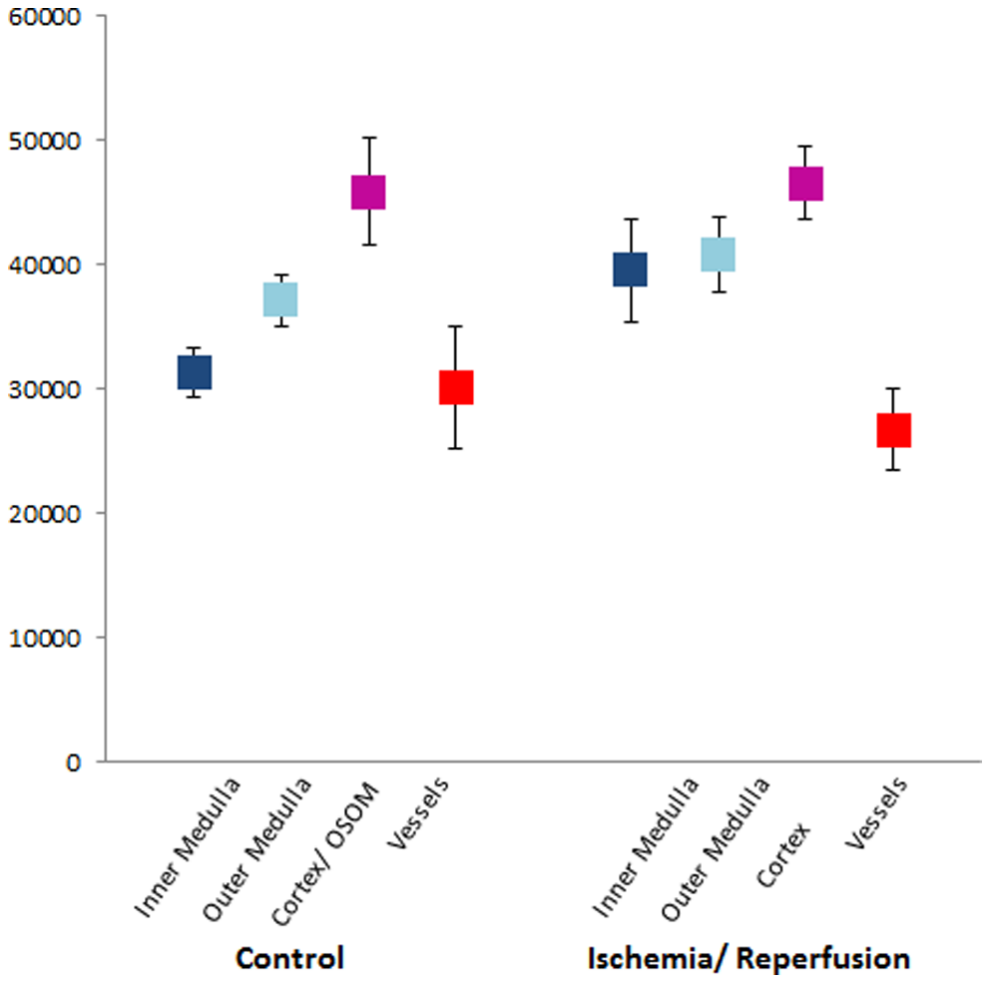
Mean Gray Values of Kidney Compartments. Mean value and standard deviations of phase gray values of the respective compartment. Note that OSOM and cortex were assessed together in healthy kidneys and OSOM and ISOM together in I/R kidneys. OSOM – outer stripe outer medulla ISOM – inner stripe outer medulla.

**Figure 4:**
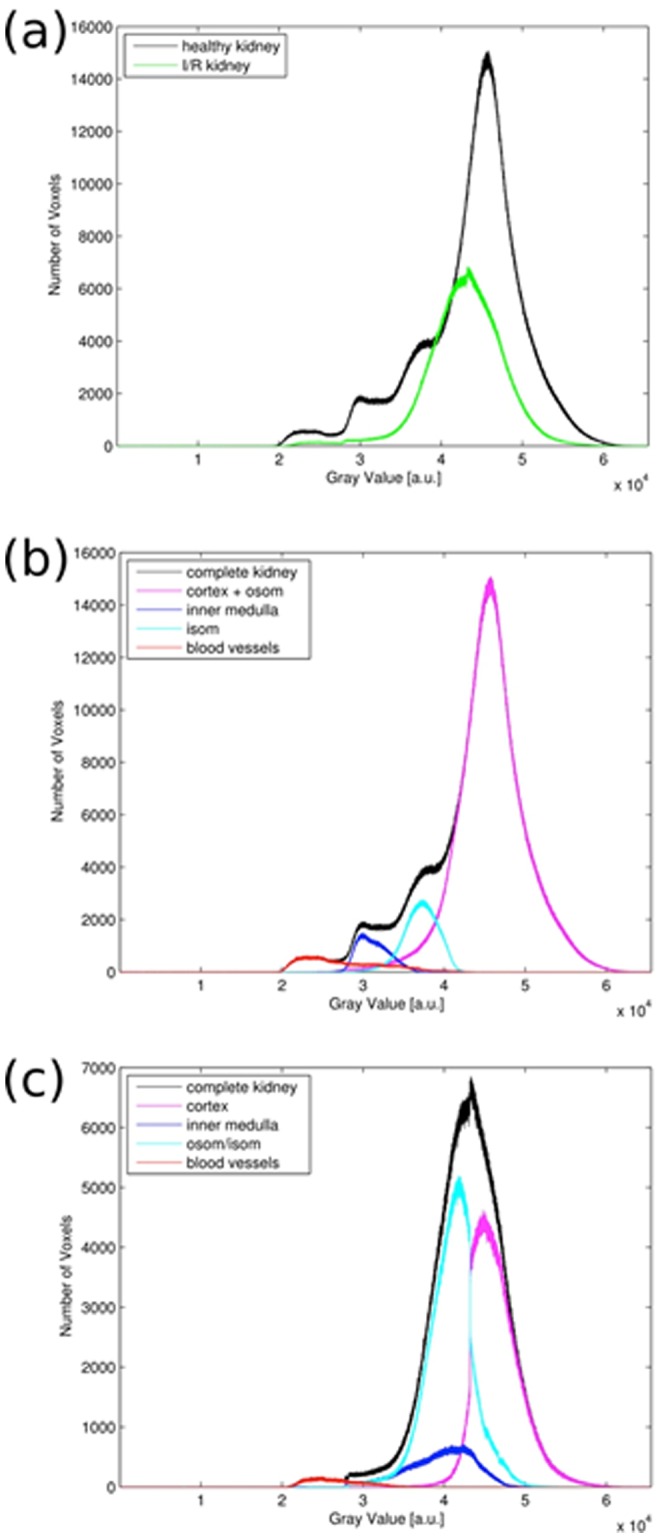
Volumetry of Healthy and I/R Kidney. Gray values are proportional to electron densities. (a) Histogram for both complete kidney volumes in comparison. (b) Histogram of healthy kidney and the contributions of the different compartments. (c) Histogram of post-ischemic kidney and the contributions of the different compartments. OSOM – outer stripe outer medulla; IS – inner stripe.

For a quantitative comparison, semi-automated segmentation and volumetry of a control and an I/R kidney was performed (**Table 1**). In the control kidneys, the different kidney compartments showed a distinct and substantial contrast (p<0.01) (**Figure 3**), whereas in I/R kidneys there was no significant difference between gray values of the inner and the stripes of the outer medulla. Histogram analysis of gray values exhibited in agreement with the visual appearance a wider range of gray values in the control kidney as compared to the I/R kidney (**Figure 4a**). In the control kidney three significantly different peaks corresponding to the cortex and outer stripe of the outer medulla, inner stripe of the outer medulla and the inner medulla could be identified (**Figure 4b**). In contrast, the histogram of gray values of the I/R kidney consisted of one merged peak with considerably overlapping sub-volumes (**Figure 4c**). Ischemia-reperfusion induced an overall organ-shrinkage by more than 50% compared to the contralateral kidney. The decrease in volume was clearly dominated by the cortex and outer medulla whereas the inner medulla remained a relatively constant volume with a ratio of 0.94.

**Table 1:** Volumetric data from three-dimensional segmentation of the functional compartments of the kidneys.

	Volume ischemic kidney [mm^3^]	Volume contralateral kidney [mm^3^]	Ratio ischemic/contralateral
total	27.96	61.14	0.46
inner medulla	2.62	2.76	0.95
Cortex/outer medulla	24.90	56.96	0.49
blood vessels	0.44	2.42	0.18

The voxel size is equal to 4.22·10^−7^ mm^3^.

### Vessel Imaging

**Figure 5:**
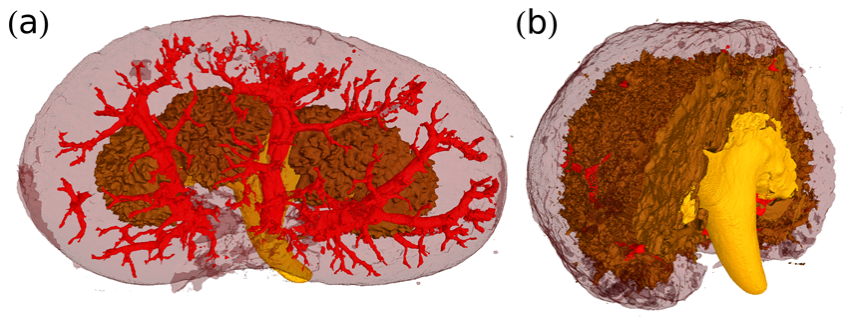
Three-Dimensional Renderings of the Renal Vasculature. (a) Healthy and (b) post-ischemic kidney from the segmented phase-contrast tomographies. The injured kidney shows a dramatic reduction of the vasculature.

GB-PCI allowed for visualization and segmentation of the renal vasculature without the use of a contrast agent. The high contrast and spatial resolution allowed to track vessel ramification from the renal hilus to the cortical branches and back to the medulla. 3D-Rendering techniques facilitated to visualize the vasculature on a single image (**Figure 5**). In I/R kidneys a dramatic reduction of vascular structures compared to the contralateral kidney could be observed and quantified (ratio 0.18).

### Correlation to Histology

**Figure 6:**
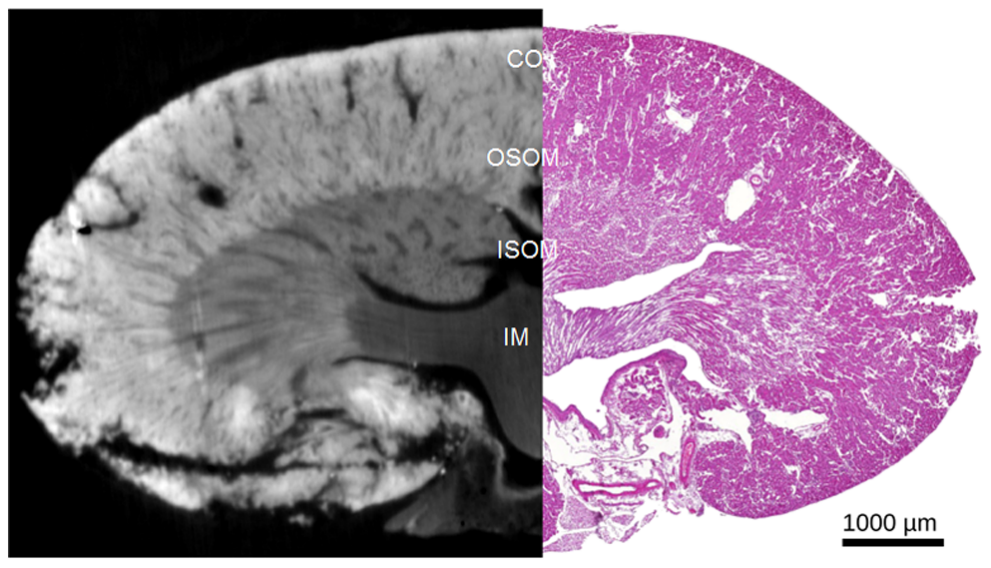
Comparison of a HE-Stained Histological Slice of Control Kidney. Phase contrast images provide similar morphological information as a HE-stained slice. The different functional compartments (IM, ISOM, OSOM/CO) can be readily differentiated. CO – cortex; OSOM – outer stripe outer medulla; ISOM – inner stripe outer medulla; IM – inner medulla.

Generally no signs of gross necrosis were found in the cortex or the tubules of the I/R kidneys. The inner and outer medulla and its stripes as well as the cortex of the healthy kidney could be distinguished by their different morphological structure of nephron and the cellularity, similarly to the phase-contrast image (**Figure 6**). No protein cylinders were detected in the tubules of the control kidneys.

**Figure 7:**
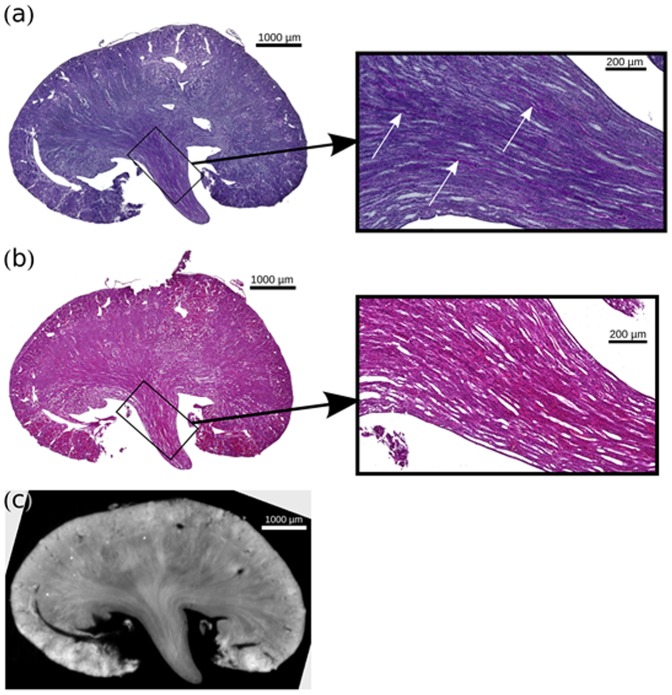
Comparison of Histological Slices of Clamped Kidney. (a) PAS histological slice of the clamped kidney. (b) HE-stained histological slice of the clamped kidney, with a similar slice of the phase-contrast volume (c). For both histology slices a respective zoom view of the inner medulla is given. Scarring and beginning tubular atrophy as well proteinaceous casts could be detected correlating with increasing medullary density in PCI.

In good agreement with the findings from the phase-contrast dataset, I/R kidneys exhibited severe tissue damage prominent in the juxtamedullary nephron (**Figure 7**). The proximal tubules showed consecutive atrophy with small lumen, cellular damage and denudation of the basement membrane with segmental shedding of both necrotic and viable epithelial cells into the tubular lumen. Focal tubules were dilated and lined with a flattened epithelium. In periodic acid-Schiff (PAS) stain, the brush border of proximal tubules was thinned or absent. Hyaline, granular casts were seen in the distal portions of the nephron and particularly prominent in the collecting ducts with focal calcification. The edematous interstitium was enlarged with a fibrillary appearance and a minimal interstitial infiltrate with lymphocytes and clustered iron macrophages could be documented. The glomeruli differed segmentally in volume with widening of Bowman's space refers to shrinkage of the glomerular capillary tuft accompanied by an apparent decrease in the number of normal glomerular lobules and by apparent loss of mesangial matrix and cells.

## Discussion

By performing volumetric and histogram analysis of the functional kidney compartments after ischemia-reperfusion damage, we demonstrate quantitative non-invasive assessment of pathophysiological processes with GB-PCI. Day 18 after unilateral clamping was chosen as an appropriate time point to assess the effects of acute kidney injury, when advanced remodeling and repair processes are highly active [31]. We chose Balb/C nu/nu mice, because the nude mice variant is more susceptible to renal ischemia-reperfusion injury than their wild-type counterparts [31]. This is in line with recent studies on the pathophysiological role of B-cells and the protective effects of regulatory T-cells in ischemia-reperfusion injury. GB-PCI can be translated from synchrotron to more accessible experimental laboratory based conditions [12]. Other studies performed at lab-based GB-PCI-settings confirmed an increase in soft-tissue contrast and a gain of complementary information for example in arteries [29] and human breast tissue [26] despite the lower density- and spatial resolution compared to synchrotron experiments. We anticipate a similar benefit for imaging of renal tissue. The feasibility of in-vivo studies with GB-PCI on small animals with acceptable radiation dose has been shown [32] for projection-based imaging at a dedicated phase-contrast and dark-field small-animal scanner. A study on dose-compatible small-animal CT imaging is ongoing. Translation to a clinical setting is still dependent on further technological developments, such as larger gratings to cover a larger field of view. Optimization of the technique in terms of acquisition time and radiation dose, for example with alternative phase extraction methods such as fourier-based single-shot imaging [33]–[35] or iterative reconstruction algorithms [35] is ongoing research.

### Diagnostic Impact of GB-PCI

MR-Imaging studies investigating acute kidney injury observed indirect changes such as alteration of the cortical and medullary T2-relaxation-time or reduced perfusion [36]–[39]. A MR-technique quantifying tissue anisotropy as an indirect measure of tubular integrity is Tensor Imaging, commonly based on diffusion (DTI) [7], [40]–[42] or as described recently on susceptibility (STI) [43]. However MRI techniques are limited in spatial resolution due to the magnetic field (in the latter reference to 55 µm). Also above mentioned MR-techniques only indirectly depict pathophysiologic changes, while GB-PCI directly assesses morphological changes of the medulla, albeit *ex vivo*. STI also still suffers from limitations that have to be overcome before translation to in-vivo scenarios, i.e. long acquisition times and necessary sample rotation relative to the magnetic field, because datasets acquired for at least 6 different orientations are necessary.

This proof-of-principle experiment clearly demonstrates the applicability of GB-PCI in renal research and pathology: Highly resolved GB-PCI using synchrotron radiation can be used to three-dimensionally examine structural changes caused by parenchymal renal disease on the level of a few micrometers facilitating volumetry. Histological sectioning only provides two-dimensional information with inevitable destruction of the sample.

After acute kidney injury we observed a significantly decreased discrimination of the different functional compartments in GB-PCI, which are presumably related to pathophysiologic changes. Usually the pathway of tubular atrophy conceptualized from morphological studies of diseased kidneys involves simplification of epithelial structure progressing to autophagy and apoptosis accompanied by marked thickening of tubule basement membranes [44]. Furthermore failed re-differentiation of the epithelial cells leads these abnormal tubules to develop thick basement membranes, autophagy and apoptosis [44]. Paracrine signaling gives rise to inflammation and finally fibrosis [2]. This evolving tubulointerstitial remodeling process may increase the average electron density particularly of the medullary compartments, and thus cause the blending-in of measured gray values, which was particularly evident for the inner and outer stripe of the outer medulla.

Interestingly, we could observe significant shrinkage of the cortex and the outer medulla. The latter forms the critical zone in ischemic injury [45]–[48]. Our visual and volumetric data directly correspond to the current pathophysiological knowledge of acute kidney injury. The earliest pathophysiological description of acute kidney injury were made by Reinhard in 1850 [49], who noted that the kidneys of patients dying from “the acute Bright's disease” had a hyperemic medulla with a pale cortex. Many investigators have demonstrated the concept of a decreased medullary blood flow as a consequence of medullary congestion [45]–[48]. Furthermore upregulation of pro-inflammatory molecules and leukocyte infiltration are most commonly observed in the outer medulla [45]. While the volume of the medulla is usually preserved, cortical atrophy follows the obstruction of the collecting ducts as described above [50], [51] We must acknowledge that in control specimen the discrimination of the outer stripe of the outer medulla from the cortex and in I/R kidney discrimination of the inner stripe of the outer stripe an inner stripe was not possible by means of gray values. The density resolution, i.e. the ability to differentiate between differences in electron density in the different tissues, can be in principle improved by the use of grating-structures with finer bars, i.e. smaller periods.

Further studies may explore the impact of the dark-field contrast, in which signal strength is determined by small-angle scattering of x-rays at microstructures that can lie on a scale below the spatial resolution of the imaging system [52], [53] thus revealing structural information that is inaccessible for transmission and phase-contrast images [32]. We have observed decreased scattering at tubular interfaces in injured kidneys, similar to what can be seen in pulmonary emphysema [25], [54] [55]. The scattering may theoretically serve as a quantifiable indirect injury marker. The absorption cross-section was inferior to phase-contrast imaging in depicting the morphological changes.

These morphological findings can be generally observed on histopathological slices, which provide abundant information on a microstructural level. However, this technique is destructive and provides only two-dimensional information on single slices. In contrast, the GB-PCI provides valuable volumetric information while maintaining an intact sample.

Furthermore GB-PCI allows for non-enhanced 3D-imaging of the renal vessels by using semi-automated segmentation of the vessel data. This is of particular interest, as many patients who would undergo renal imaging suffer from chronic kidney disease and are thus at high risk for either contrast induced nephropathy or nephrogenic systemic fibrosis. The observed differences in soft-tissue contrast were obtained without the use of any contrast agent. One must acknowledge that gray-value based differentiation between arteries and veins is not possible, but only by visual selection. The applicability of contrast agents in phase-sensitive x-ray imaging is still under investigation. Besides iodinated contrast agents, the main focus is currently on contrast media with x-ray scattering properties for dark-field imaging such as microbubbles, which can be also administered in patients with renal insufficiency [56].

### Challenges and Limitations

One challenge to overcome is the optimization of automatic segmentation of the kidney compartments, as this is a potential error source. The outer stripe of the outer medulla could be identified in the healthy kidney by means of the different tissue texture but not gray value and was virtually not distinguishable from the inner stripe in the I/R kidney. So quantitative comparison of the cortex and the outer medulla could not reliably performed in this study. While the other functional compartments could be readily discriminated in the healthy kidney, the segmentation of the I/R kidney dataset was more prone to errors. Here the compartments could not be separated by thresholding and region growing only, so that additional manual segmentation had to be performed, introducing a potential human source for segmentation errors. For a more objective segmentation, the area of image processing and machine learning provides a wide variety of adaptive dedicated algorithms [57], [58].

Another potential source of error is the influence of formalin fixation on soft-tissue signal characteristics in phase-contrast imaging. This has only been examined for brain tissue [16] and is currently under investigation for other tissue types [19]. Security regulations at the synchrotron radiation facility did not allow for the measurement of fresh kidney samples. Thus the gray values displayed in this work are proportional to the respective electron density of the formalin fixated sample. We do not present absolute numbers of electron density or their respective conversion to phase-contrast Hounsfield units, because the values measured here may not exactly represent the values for fresh or *in-vivo* tissue because of potential effects of the fixation process.

In general, GB-PCI is still associated with high radiation doses and the presented study has not been optimized in this regard. However, alternative acquisition and post-processing techniques as well as dedicated iterative reconstruction algorithms [35] are currently being developed to reduce the necessary radiation dose [59], [60]. Furthermore, the facilitated access to laboratory-based setups with polychromatic x-ray sources and a larger – however still limited - field of view, in contrast to synchrotron radiation facilities, allows for studies with an increased number of samples and larger sample sizes. It remains to be determined if similar image quality can be achieved in a potential human in-vivo setting at acceptable radiation doses.

Another limitation to this study is that the assessment of volumetric data is subject to particular biases. First of all, the imaged kidney samples were cut in half after excision to allow for proper formalin fixation. This was performed similarly for both kidneys, so that the deduced ratios are still valid, however the absolute values would have to be doubled. Actually, our findings were in line with a previous study investigating kidney volume with ultrasound. Here a mean volume of 0.1 cm^3^ was determined for wt/wt mice [61]. Another study investigated kidney volume of CD1-mice. Here a mean volume of 0.2 cm^3^ was determined [62]. If doubled, the volume of our control kidney would result in 0,12 cm^3^. Secondly, the contralateral kidney served as internal control. After unilateral renal ischemia the contralateral kidney may undergo a certain hypertrophy [39], so that the observed volumetric shift may also be partly due to this process.

## Conclusions

GB-PCI with synchrotron radiation allows for noninvasive and non-destructive assessment of parenchymal kidney disease on the microstructural level and vessel architecture in three dimensions and thus serves well as a volumetric alternative to histological slicing. Since GB-PCI using conventional polychromatic x-ray tube sources has been proven successful, the value of laboratory-based GB-PCI on the investigation of parenchymal renal disease of murine and human kidney samples has to be assessed in further studies. If the density resolution achieved with polychromatic sources is sufficient to differentiate between diseased and healthy tissue, GB-PCI may serve ultimately as a non-invasive, non-contrast media enhanced method to study pathological changes of the kidney and become an alternative to current imaging methods.
